# Association of Visual Heart Score with Gross Lung Pathology and Histology of Hepatic and Cardiopulmonary Tissues in Cattle at Harvest

**DOI:** 10.3390/ani16081248

**Published:** 2026-04-18

**Authors:** Makenna J. Jensen, Brad J. White, Robert L. Larson, Phillip A. Lancaster, Todd G. Gunderson, Brandon L. Plattner, Justin W. Buchanan, Sierra Crisp, Randall C. Raymond

**Affiliations:** 1 Beef Cattle Institute, Department of Clinical Sciences, College of Veterinary Medicine, Kansas State University, Manhattan, KS 66506, USArlarson@vet.k-state.edu (R.L.L.);; 2 Kansas Veterinary Diagnostic Laboratory, Department of Diagnostic Medicine and Pathobiology, College of Veterinary Medicine, Kansas State University, Manhattan, KS 66506, USA; 3 Simplot Livestock Company, Boise, ID 83707, USA

**Keywords:** bovine congestive heart failure, heart score, lung deflation, protozoal cyst

## Abstract

Non-infectious cardiac failure in feedyard cattle has become more frequently diagnosed. There is limited research assessing gross and histologic lesions in grossly abnormal hearts of harvested cattle. Cases were stratified by heart score (HS) using a scale of 1–5, with 1 representing a normal heart and 5 representing severely remodeled ventricles. Cattle were evaluated for gross lesions of the heart, lung, and liver. Samples collected from each animal for histology included cardiac (n = 4), pulmonary (n = 4), and hepatic (n = 1) tissues. Histologic evaluation scored cardiac and hepatic fibrosis and necrosis, embedded myocardial protozoal cysts (EMPCs) were quantified, and pulmonary lesions were categorized based on histologic patterns. Of 103 cases, 40 had normal HSs (NHSs) (1 or 2), and 63 had abnormal HSs (AHSs) (3, 4, or 5). There were 64 cases with normal lung deflation scores, and 39 cases with abnormal lung deflation scores. At least one cardiac section contained EMPCs in 67 cases. Cattle with abnormal lung deflation scores were more likely to have an AHS (0.76 ± 0.07, *p* ≤ 0.01) compared with cattle with normal deflation scores (0.52 ± 0.06). Cattle with EMPCs present in at least one cardiac section were more likely to also have an AHS (0.73 ± 0.05, *p* ≤ 0.1) compared with cattle without EMPCs (0.39 ± 0.08). No histological findings for the lungs or liver were associated with abnormal heart score; however, lung deflation and EMPCs were associated with abnormal heart score.

## Simple Summary

Interest in the cardiac health of feedyard cattle has gained momentum over the past decade. With non-infectious cardiac failure becoming more frequently diagnosed, the objective of this study was to evaluate potential associations between the gross pathology and histopathology of the lungs, heart, and liver of cattle at harvest. The lungs and liver of cattle were evaluated for gross pathology, with the lungs scored for failure to deflate and the hearts scored for cardiac ventricular enlargement. Samples of the lungs, heart, and liver were collected for histologic evaluation. Both heart and lung scores were dichotomized into normal and abnormal scores for statistical evaluation. Cases where lungs failed to deflate had a higher probability of an abnormal gross heart score. Cattle with the presence of embedded myocardial protozoal cysts had a higher probability of an abnormal heart score. No specific histologic lesions in the lung, heart, or liver were associated with abnormal heart score or specific subcategories of pulmonary lesions. The importance of ongoing research on cardiac health in cattle is emphasized in this study, as some results were unexpected and warrant further investigation.

## 1. Introduction

Prior to the early 2000s, non-infectious cardiac failure in cattle had a low reported incidence, was more commonly seen at elevated altitudes, and was mostly considered part of the syndrome known as high-mountain or high-altitude disease [1]. However, in more recent years, bovine congestive heart failure (BoCHF) has become more frequently diagnosed at lower elevations, indicating that elevation is not always a significant factor [1,2]. Clinical signs of BoCHF are variable but include dyspnea, edema of the brisket and ventral abdomen, jugular venous distention, moist cough, depression or inappetence, exercise intolerance, and abducted elbows [3,4,5,6,7,8]. Bovine right-sided congestive heart failure may be initiated by hypoxia (e.g., high elevation, pneumonia, obesity, heat stress, or lungworm infection), which causes vasoconstriction and resultant pulmonary hypertension [2]. Increased pulmonary vascular pressure results in increased right ventricular afterload, causing stress on the cardiac walls. In turn, right ventricular hypertrophy is caused by remodeling and degeneration of the extracellular matrix (ECM) and myocardial interstitial connective tissue and collagen [2,9]. Sustained periods of myocardial injury and remodeling eventually lead to a reduced compensatory capability of the cardiac wall, and clinical congestive cardiac failure in the individual; morphologic lesions only accompany the chronic phases of injury [9].

Gross morphologic cardiac lesions, especially ventricular enlargement, are described using a scale from 1 to 5. A gross heart score (HS) of 1 indicates a normal heart without ventricular enlargement; an HS of 5 indicates a severely remodeled heart with markedly enlarged and distorted ventricles [10]. A recent study documented cardiac enlargement and misshapen ventricles (CEMVs) in feedlot cattle mortalities, but cattle with evidence of congestive heart failure were only a subset of these cases. In the forementioned study, cases of BoCHF were defined as having CEMVs with a congested liver and two or more effusions such as pericardial, pleural, or peritoneal effusions [10]. Another study of feedyard mortalities, using the same definition for BoCHF, found that 35.7% of mortalities had lesions associated with cardiovascular disease (BoCHF, heat stress, right or left ventricular concentric hypertrophy, anemia, endocarditis, myocarditis, nutmeg liver, or pericarditis) with 15.3% of those being BoCHF [11]. Few studies have looked at the changes in cardiac morphology of cattle at harvest, but a 4.14% incidence of an HS of 4 or 5 has been described in a sample population of 32,763 commercial feedyard cattle [3]. Further studies have shown an association between CEMVs and failure of lungs to deflate after death in harvested cattle; a hypothesis is that necrosis and fibrosis of the lungs associated with chronic congestive heart failure prevents normal deflation [12]. Failure of the lungs to deflate could be associated with pulmonary hypertension either preceded or followed by cardiomegaly. The goal of this study was to determine the relationship between HS and histologic architecture of the heart, lung and liver in feedlot cattle at harvest.

Three main objectives were defined for this study. The first objective was to determine if gross lung deflation or histologically confirmed pneumonia were associated with HS. The second objective was to determine if HS at harvest was associated with cardiac fibrosis, necrosis, or cardiomyofibrillar abnormalities. The third objective was to determine if an abnormal HS at harvest was associated with hepatic fibrosis, necrosis or underlying hepatocellular abnormalities. We hypothesized that the presence of chronic pulmonary lesions, abnormal histologic cardiac lesions, or the presence of abnormal histologic hepatic lesions would be associated with increased or abnormal heart scores.

## 2. Materials and Methods

A cross-sectional observational study was performed that evaluated the association of HS with gross lung pathology and histopathology of the cardiopulmonary and hepatic systems. This study was exempt from approval by the Institutional Animal Care and Use Committee because all data and sample collection occurred after death. USDA regulations for post-mortem cattle at slaughter, including the Food Safety and Inspection Service (FSIS) and Office of Laboratory Animal Welfare (OLAW) guidelines, were followed.

### 2.1. Gross Evaluation

**Figure 1:**
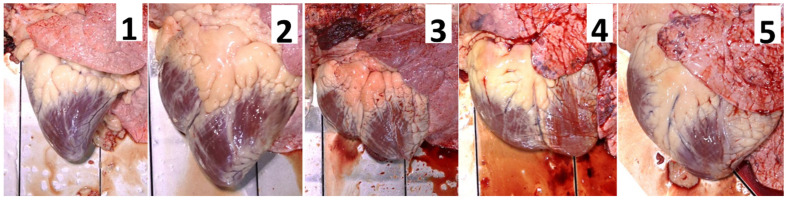
Pictured above starting on the left side is an example with a heart score (HS) of (**1**). The right ventricle can be seen on the left side of the image and is tight with no rounding and the heart comes to a V-shaped apex. Moving to the right, hearts with HSs of (**2**) and (**3**) have increasingly enlarged right ventricles, as seen by the rounding on the left side of the image. A heart with an HS of (**4**) has a characteristic W-shape. The picture on the right side is a heart with an HS of (**5**) with severe cardiac changes, the loss of a tight right ventricle, as seen by rounding and the loss of the apex, resulting in a U-shape.

**Figure 2:**
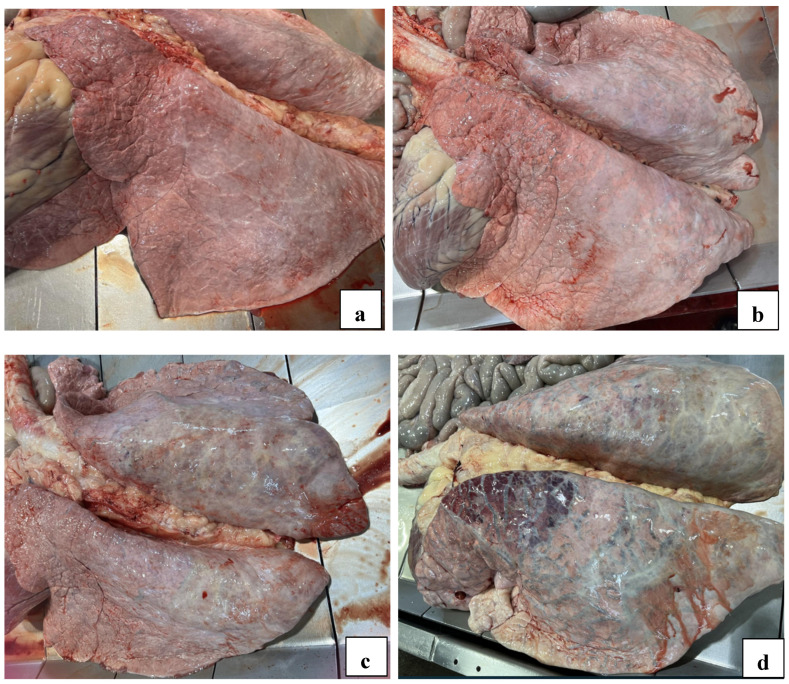
(**a**): Normal deflated lungs; (**b**): mild failure to deflate; (**c**): moderate failure to deflate; (**d**): severe failure to deflate. The margins of the normal lungs are defined, sharp, and they are completely collapsed with no turgidity or stiffness of the tissue. With increased severity, the margins of the lungs become rounded. Increased severity is determined by increased expansion and turgidity of the lung tissue.

Three evaluators (R. Raymond, J. Buchanan, and S. Crisp) collected samples from a targeted cross-section of cattle at harvest that had passed USDA antemortem inspection (n = 121), which were stratified by HS: 1 = normal, 2 = mild ventricular enlargement, 3 = moderate ventricular enlargement, 4 = severe ventricular enlargement, and 5 = severe ventricular enlargement and flaccid, as summarized in Figure 1 [10]. Individual animals were evaluated for gross lesions of the heart, lung, and liver including failure of lungs to deflate, pulmonary edema, interstitial or bronchopneumonia, and liver abscesses. Assessments of cavitary effusions were not recorded. All cases received a gross pulmonary deflation score of either normal/deflated, mild failure to deflate, moderate failure to deflate, or severe failure to deflate. Normal lungs were defined as completely collapsed and pliable with no turgidity, mild to moderate failure to deflate had increased expansion and turgidity, and severe failure to deflate were fully expanded lungs with complete turgidity and stiff tissues (Figure 2). Each case was assigned a gross lung lesion description (bronchopneumonia, interstitial pneumonia, or pleuritis) and the percent of lung affected was estimated, with categories ranging from normal (<5% lesions), 5–15% lesions, 15–30% lesions, 30–50% lesions, and 50%+ lesions. Nine tissue samples from each animal were collected for histopathologic evaluation from the same anatomic location for each animal: four cardiac [septum, papillary muscle, right ventricular free wall (RVFW), and left ventricular free wall (LVFW)], four pulmonary [right and left cranioventral lobes, and right and left caudodorsal lobes], and one representative liver section from the anterior surface near the midline.

### 2.2. Histopathology

All samples collected for histopathology were approximately 1 cm^2^ in size, which were fixed for at least 24 h in 10% neutral buffered formalin. Once the samples arrived at the laboratory, they underwent processing for serial dehydration and paraffin embedding. Each sample was then sectioned into 4 µm sections and stained with hematoxylin and eosin using a Leica ST5010 Autostainer XL (Leica Biosystems, Deer Park, IL, USA). The prepared samples for histopathology were evaluated by a board-certified veterinary pathologist (B. Plattner) and veterinarian (T. Gunderson) who were cross-trained and supervised by the same pathologist. The evaluators were blinded to all gross lesions including gross heart scores, lung lesion scores, and liver evaluation. All histology evaluations were finalized by the pathologist.

Histologic heart and liver sections each received an autolysis score defined as 0 (no autolysis), 1 (mild autolysis and increased scattered bacterial overgrowth), 2 (moderate autolysis, loss of nuclear and cellular details, and moderate bacterial overgrowth), or 3 (marked autolysis, cell and nuclear morphology difficult or impossible to discern, and severe bacterial overgrowth). Cardiac and hepatic samples were assigned a fibrosis score of 0 (normal), 1 (mild subendocardial or interstitial fibrosis with increased loose to compact fibrous tissue), 2 (moderate compact interstitial fibrosis with loss and replacement of cardiomyocytes and/or hepatocytes), or 3 (marked patchy regional interstitial fibrosis and replacement or bridging fibrosis/fibroplasia); and a necrosis score of 0 (normal, no necrosis), 1 (mild individual scattered cells showing coagulative necrosis), 2 (moderate patchy coagulative necrosis), or 3 (marked patchy and bridging coagulative necrosis). Additionally, cardiac samples were scored based on the number of embedded protozoal cysts, which was defined as rare (0–1 cyst per tissue section, approximately 400 mm^2^), few (2–10 cysts per tissue section), moderate (>10 cysts per tissue section), or severe (>50 cysts per tissue section). The pulmonary tissue sections were categorized based on the most prominent pneumonia pattern: bronchopneumonia (BP), interstitial pneumonia (IP), or other (OTH), which was taken from Feitoza et al. and modified (Table 1) [13].

### 2.3. Data Categorization

Gross heart scores were grouped into normal (HS of 1 or 2) (NHS) or abnormal (HS of 3, 4, or 5) (AHS). The lung deflation score was categorized as normal (deflated) or abnormal (mild, moderate, and severe failure to deflate). Heart scores of 3, 4, or 5 were considered abnormal to increase the sensitivity for associations with histologic cardiac lesions. Similarly, the lung deflation score was grouped with the abnormal category consisting of mild, moderate, and severe failure to deflate to increase the sensitivity for associations with histologic pulmonary lesions. Gross lung lesion scores (percent lung affected with lesions) were categorized as normal (<5% pathology) or abnormal (5–15%, 15–30%, 30–50%, and 50%+ pathology). Cardiac and hepatic fibrosis and necrosis were categorized as normal (0, 1) or abnormal (2, 3). Protozoal cyst scoring of the heart was dichotomized into rare (none or rare) or present (few, moderate, or severe).

Using the pneumonia pattern scoring system for each of the four individual lung samples, a final case diagnosis was assigned: BP, exudate or proliferative IP, bronchopneumonia with an interstitial pattern (BIP), lymphoplasmacytic interstitial pneumonia (LIP), or other (OTH). Chronicity was also assessed in each lung section, and if any of the four lung samples had detectable fibroplasia, the case was considered chronic. In order to be considered BP, one or more of the samples had to show BP characteristics but with no other pneumonia features except for LIP or OTH. For a diagnosis of IP, one or more of the samples had to exhibit exudative or proliferative IP but with no other pneumonia patterns except for LIP or OTH. If one or more lung sections showed BP and one or more other lung sections of the same case showed exudative or proliferative IP, the diagnosis was BIP. If one or more lung sections exhibited chronic LIP and no other pattern other than OTH, they were diagnosed as LIP. Cases diagnosed as OTH could only have characteristics of OTH present. OTH was defined as edema, congestion, atelectasis, emphysema, or no lesions.

### 2.4. Analysis

A series of logistic regression models were constructed in Rstudio (version 4.5.0, Boston, MA, USA) to evaluate the probability of an AHS. Each independent variable of interest was first modeled in a univariate logistic regression using a logit link function. Type III likelihood ratio tests were performed using the car::Anova() function to provide *p*-values for each predictor [14]. Estimated marginal means with pairwise contrasts were calculated with the emmeans package to present results on a probability scale. Contingency tables were also generated to visualize raw counts of outcomes across predictor categories.

Independent variables included categorized covariates evaluating lung pathology (gross deflation, percent affected, and pneumonia diagnosis), cardiac lesions (fibrosis, necrosis, and embedded myocardial cysts at the animal level and sample level), hepatic lesions (fibrosis and necrosis), and pneumonia pattern scoring from each lung lobe (RCv, RCd, LCv, and LCd). A separate binary logistic regression model, fitted using glm() with a binomial error distribution and logit link function, was then used to assess associations between AHS and pneumonia patterns with an interstitial component (AIP, CIP, CBIP, and CLIP) or lesion chronicity (acute/chronic). The IP component and lesion chronicity were fixed effects, and an interaction term between the two was included in the model.

Predictors were evaluated in univariate models and then placed into a final multivariate model if the *p*-value was ≤0.3. As with the univariate models, the multivariate model was summarized using coefficient estimates, standard errors, Wald tests, and Type III likelihood ratio tests. Pairwise comparisons of predicted probabilities across factor levels were obtained using emmeans. Covariates included in the multivariate model included gross lung deflation and protozoal cysts at the animal level and sample level, and pneumonia pattern scoring for each lung lobe. Gross lung deflation and protozoal cysts were evaluated for each animal, but aliased coefficients and multicollinearity precluded analysis of protozoal cysts and pneumonia pattern scoring using multiple samples from the same animal. Predictors with a *p*-value of ≤0.05 were utilized in the final model that included gross lung deflation and protozoal cyst categories for each animal. Co-linearity between gross lung deflation and the presence of protozoal cysts was assessed using a variance inflation factor (VIF).

## 3. Results

**Figure 3:**
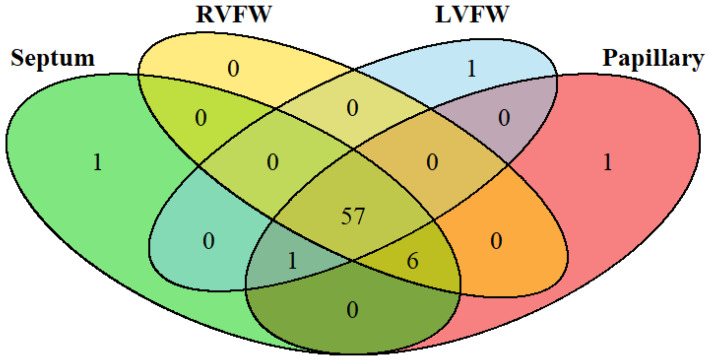
Number of cattle with embedded protozoal cysts present, and which cardiac samples they were observed in. Four heart sections were collected and reviewed histologically including the septum, right ventricular free wall (RVFW), left ventricular free wall (LVFW), and papillary muscle. Each sample was given a score for embedded myocardial protozoal cysts (EMPCs), defined and then categorized into rare or present (few, moderate, or severe). Most animals with EMPCs present showed EMPCs in all four sections of the heart.

**Figure 4:**
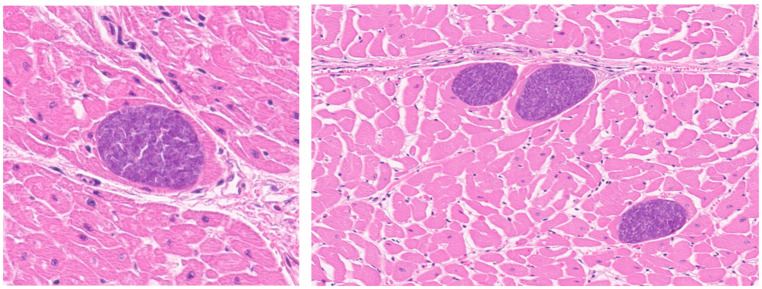
Embedded myocardial protozoal cysts in H & E-stained sections. These images show embedded myocardial protozoal cysts within cardiac muscle. They are the basophilic staining, circular structures.

Out of 121 cases, 15 had missing or incomplete tissue sample sets, one had an incomplete animal ID, one had a missing lung deflation score and lung percent affected score, and one was diagnosed histologically with infectious pericarditis; these were excluded, leaving a total of 103 cases for further evaluation. Forty cases had an NHS and 63 had an AHS (Table 2). Sixty-four cases had normal lung deflation scores, while 39 had abnormal scores. Of the 39 cattle with abnormal lung deflation scores, 33 had ≤5% gross lung lesions. Out of the total 103 cases, eight had significant cardiac fibrosis in at least one heart section, and only four cases had significant necrosis in at least one of the heart sections (Table 3 and Table 4). Sixty-seven cases had embedded protozoal cysts within at least one of the four cardiac sections. Of the 67 cases with embedded protozoal cysts, 57 had protozoal cysts in all four cardiac sections (Figure 3 and Figure 4). Histopathology of the liver revealed two cases with abnormal scores for both fibrosis and necrosis, one case with an abnormal fibrosis score, and one case with an abnormal necrosis score. After final pneumonia diagnosis, there were five acute BP (ABP), five chronic BP (CBP), 12 acute IP (AIP), two chronic IP (CIP), three chronic BIP (CBIP), 75 chronic LIP (CLIP), and one OTH cases (Figure 1). Of the 39 cases with failure of lungs to deflate, the histologic diagnosis included 7 AIP, 2 ABP, 3 CBIP, 4 CBP, 2 CIP, and 21 CLIP cases.

Independent variables with a *p*-value ≤ 0.3 that were included in the multivariate regression model were gross lung deflation, the presence of embedded protozoal cysts at the animal level, and lung histopathology at the sample level. The lung histopathology had aliased coefficients indicating perfect multicollinearity between two or more of the lung samples. Therefore, lung histopathology was removed from the multivariate model.

The final multivariate model included gross lung deflation and protozoal cysts at the animal level with an outcome of AHS. Cattle with lungs that failed to deflate had a higher probability of an AHS (0.76 ± 0.07, *p* ≤ 0.01) than cattle with normally deflated lungs (0.52 ± 0.06). Cattle with cardiac embedded protozoal cysts at the animal level were more likely to be have an AHS (0.73 ± 0.05, *p* ≤ 0.1) than cattle without protozoal cysts present (0.39 ± 0.08). The VIF for lung deflation and embedded protozoal cysts at the animal level was 1.06, suggesting that both factors were independently associated with AHS.

## 4. Discussion

Cattle affected with pneumonia or had experienced pneumonia previously often have increased and irreversible pulmonary fibrosis, which is a normal physiologic or healing response to severe or persistent inflammation [15]. Interstitial pulmonary fibrosis causes damage to the alveolar walls, a decreased air exchange capacity, decreased lung distensibility, and pulmonary hypertension, leading to increased right-sided cardiac afterload and right-sided cardiac hypertrophy [15]. In this sample set, lungs with abnormal lung deflation scores had minimal gross lesions and a wide variety of subcategories of pneumonia histologically. This finding warrants further investigation but suggests that our current methods for gross and histologic evaluation of pulmonary tissue do not provide an adequate explanation for the proposed pathophysiologic pathway of cardiac remodeling due to microscopic or gross pulmonary changes.

Where there are disagreements between gross and histologic diagnoses of pneumonia patterns in cattle, histopathology is considered a more accurate methodology [16]. Differences between the percent of lungs exhibiting pulmonary lesions and the number of cases diagnosed with CLIP at histologic evaluation are notable. CLIP lesions may not be readily observed grossly; however, the increased inflammation in the pulmonary interstitium may affect the ability of lungs to collapse. Recent work has shown a prevalence of 12.6% for CLIP in feedyard mortalities and concluded that based on the distinct lesions (especially from exudative or proliferative IP patterns), CLIP represents a different pathogenic process than IP [13]. While 73% of cattle in this study were diagnosed with CLIP histologically, it is not a representative population of all feedyard cattle because the sample was first stratified by HS, and thus should not be directly compared to prevalences in feedyard cattle populations (Table S1).

A positive association between the presence of embedded protozoal cysts within the heart and AHS was noted in this study. These protozoan parasites are commonly observed in bovine hearts and are typically presumed to represent *Sarcocyst* spp. Often observed in the absence of significant inflammation, these cysts are typically reported as an incidental finding. The association between AHS and sarcocyst embedment in cardiac tissue warrants additional consideration of the effect of the parasite on cardiac remodeling. Canids and felids are the most common definitive hosts for *Sarcocyst* spp., but some species transmitted by canids may be more pathogenic [17]. *Sarcocyst* species identified in cattle include *S. cruzi*, *S. hominis*, *S. bovifelis*, *S. bovini*, *S. hirsuta*, and *S. heydorrni*. Of these, *S. hominis* and *S. heydorrni* have been shown to be zoonotic [18,19]. *S. cruzi* is the most common species found in cattle, with a prevalence of 56.5% of carcasses at harvest, followed by *S. hominis* at 21% [18]. Species identification was not performed during this study.

Clinical sarcocystosis leads to anorexia, weight loss, abortion, hyperexcitability, and death [17,18]. Subclinical infection has been reported in up to 100% of harvested cattle; in this study, 65% of carcasses had at least some protozoal cysts present [18,20,21,22,23,24,25,26,27,28]. Sarcocystosis is only diagnosed by histopathology and is typically unrecognizable clinically. A small subset of these cases can have more widespread disease, known as bovine eosinophilic myositis (BEM). These gross lesions appear as gray, white, or green foci that lead to condemnation at slaughter. Disseminated inflammatory lesions have been found in the heart, tongue, diaphragm, esophagus, and skeletal muscle [17,19]. Cases condemned for BEM were not documented in this study.

Cattle infected with *S. cruzi* are commonly noted to have interstitial and perivascular mononuclear cell infiltration in various organs including the kidneys, liver, and lungs [17]. Given the histologic lesions commonly seen in these animals, we hypothesized that inflammation or fibroplasia may contribute to decreased blood-gas exchange, and possibly pulmonary hypertension or right-sided cardiac hypertrophy [15]. We occasionally observed protozoa embedded within the interstitium, adjacent to or around vessels, and within structures of the conduction system (Purkinje fibers and bundle branches). It is plausible that parasite-associated inflammation may contribute to cardiac injury and remodeling, which may be a cause of an AHS. However, a limitation of this study is that protozoal-associated inflammation was not significantly increased in animals with evidence of cardiac remodeling, so further research studying the association of protozoa, fibroplasia, and inflammation associated with AHSs may be warranted.

Cardiac fibrosis has been reported in bovine hearts and is usually considered either an early and more mild reactive fibroplasia, or a more severe and extensive chronic form known as replacement fibrosis [8,9]. Based on the human literature, reactive fibrosis can be seen as interstitial fibrosis, defined as a dissection of collagen between myocytes, and perivascular fibrosis noted specifically around interstitial and coronary blood vasculature. Replacement fibrosis is visualized in areas of cardiomyocyte loss after necrotic events and is considered chronic granulation or scar tissue, where fibroblasts replace degenerate and lost cardiomyocytes and become the majority cell type in the cardiac wall [4,9]. Rigidity in a structurally and functionally normal heart can be attributed to a predominance of type 1 collagen. Loss of type 1 collagen due to injury leads to decreased tensile strength and subsequent ventricular dilation. Unlike cardiomyocytes, fibroblasts are capable of re-entering the cell cycle, which contributes to hyperplasia or fibrosis [29]. In humans, cardiac remodeling with fibrosis has been documented to lead to diastolic dysfunction, pulmonary hypertension, arrhythmias, and sudden death [9].

The majority of cardiac fibrosis and necrosis scores were observed to be zero, i.e., most cattle were found to have very mild cardiac changes. Cardiac fibrosis has been previously studied in cattle with clinical signs of BoCHF. Krafsur et al. described feedyard mortalities of cattle with clinical signs of BoCHF as having significant perivascular, interstitial, and replacement fibrosis present; two case–control cattle out of 20 also had notable interstitial fibrosis and mild perivascular and replacement fibrosis [4]. In a separate study, Krafsur et al. noted that cattle symptomatic for BoCHF had severely atrophied hepatic cords, centrilobular necrosis, and centrilobular fibrosis [4]. However, the current study saw very few cattle with hepatic fibrosis or necrosis. Cattle with hepatic fibrosis and necrosis present had very mild changes. This was unexpected as we hypothesized that histologic hepatic lesions such as necrosis and fibrosis would be associated with AHSs. Additional work is warranted to understand the relationship between cardiac or hepatic fibrosis and necrosis, cardiac function, and cardiac ventricular wall remodeling.

## 5. Conclusions

In this study we observed a significant positive association between abnormal gross lung deflation and AHS; however, we were unable to consistently document specific histologic lesions in the lung, heart, or liver associated with AHS, or specific subcategories of pulmonary lesions including BP, BIP, IP, or CLIP. An association with cardiac and hepatic fibrosis or necrosis and AHS was not demonstrated. The presence of embedded myocardial protozoal cysts was positively associated with AHS.

## Figures and Tables

**Table 1:** Pneumonia pattern scores.

Pattern Score	Major Lung Pattern	Type	Age	Pneumonia Type
1	Acute exudative interstitial pneumonia (hyaline membranes, septal necrosis)	IP	Acute	AIP
2	Subacute proliferative interstitial pneumonia	IP	Acute	AIP
3	Chronic fibrosing interstitial pneumonia (may include bronchiolitis obliterans)	IP	Chronic	CIP
4	Chronic lymphoplasmacytic interstitial pneumonia	LIP	Chronic	CLIP
5	Acute fibrinosuppurative bronchopneumonia	BP	Acute	ABP
6	Subacute proliferative and histiocytic bronchopneumonia	BP	Acute	ABP
7	Chronic bronchopneumonia with fibrosis (may include bronchiolitis obliterans)	BP	Chronic	CBP
8	Bronchiectasis and bronchiolectasis (caseonecrotic bronchopneumonia)	BP	Chronic	CBP
9	Fibrinous pleuritis and pleuropneumonia	BP	Acute	ABP
10	No lesions/edema/congestion/atelectasis/emphysema	OTH		OTH

Four lung sections (right and left cranioventral and right and left caudodorsal lobes) were submitted for histopathologic evaluation. Each section was given a pattern score based on the major lung lesions present.

**Table 2:** Number of cases with gross pathological lesions by heart score.

Heart Score	HS1	HS2	HS3	HS4	HS5
**Number of Cattle**	21	19	23	25	15
**Gross Lung Deflation**					
Normal (deflated)	20	11	16	11	6
Abnormal (mild, moderate, or severe failure to deflate)	1	8	7	14	9
**Percent Lung Affected with lesions**					
Normal (<5% lesions)	21	17	22	22	14
Abnormal (≥5% lesions)	0	2	1	3	1

**Table 3:** Number of cases for each fibrosis score by sample location.

Fibrosis Score	Septum	Papillary Muscle	Right Ventricular Free Wall	Left Ventricular Free Wall	Liver
**0**	49	44	57	64	78
**1**	52	55	44	39	18
**2**	2	4	2	0	3
**3**	0	0	0	0	1
**NA**	0	0	0	0	3

**Table 4:** Number of cases for each necrosis score by sample location.

Necrosis Score	Septum	Papillary Muscle	Right Ventricular Free Wall	Left Ventricular Free Wall	Liver
**0**	55	49	49	56	73
**1**	47	53	53	46	23
**2**	1	1	1	1	4
**3**	0	0	0	0	0
**NA**	0	0	0	0	3

This table shows the number of cases that had fibrosis or necrosis with each of the three scores for heart and liver sections before they were dichotomized into normal (0 and 1) and abnormal (2 and 3). Not Assessed (NA) indicates cases with missing liver samples.

## Data Availability

The data used in this research was provided by cooperating entities and are not publicly available due to confidentiality agreements.

## Supplementary Materials

The following supporting information can be downloaded at: https://www.mdpi.com/article/10.3390/ani16081248/s1, Table S1: Descriptive statistics of histologic lesions by heart score.

## Abbreviations

The following abbreviations are used in this manuscript:

HS	Heart score
NHS	Normal heart score
AHS	Abnormal heart score
BoCHF	Bovine congestive heart failure
CEMVs	Cardiac enlargement and misshapen ventricles
EMPC	Embedded myocardial protozoal cyst
