# Supplementary material for: Association of Visual Heart Score with Gross Lung Pathology and Histology of Hepatic and Cardiopulmonary Tissues in Cattle at Harvest
Source: Animals (Basel). 2026 Apr 18;16(8):1248. doi: 10.3390/ani16081248 (PMC13113706; doi:10.3390/ani16081248)
Supplement: Supplementary file 1 [file animals-16-01248-s001.zip › animals-4252925-supplementary.pdf]

Supplemental Table S1: Descriptive statistics of histologic lesions by heart score.

Heart Score:	HS1	HS2	HS3	HS4	HS5
**Pneumonia Patterns:**					
CLIP Diagnosis	19	16	20	13	7
ABP Diagnosis	2	0	2	0	1
CBP Diagnosis	0	0	0	3	2
AIP Diagnosis	0	1	1	7	3
CIP Diagnosis	0	2	0	0	0
CBIP Diagnosis	0	0	0	1	2
OTH Diagnosis	0	0	0	1	0
**Gross Lung Deflation:**					
Normal	20	11	16	11	6
Mild failure to deflate	1	6	3	8	3
Moderate failure to deflate	0	2	4	3	4
Severe failure to deflate	0	0	0	3	2
**Lung Percent Affected with Pathology:**					
Normal<5%	21	17	22	22	14
5-15%	0	1	0	1	1
15-30%	0	1	0	1	0
30-50%	0	0	0	0	0
50+%	0	0	1	1	0
**Cardiac Fibrosis:**					
Normal (0, or 1)	21	18	22	22	14
Abnormal (2 or 3)	0	1	1	3	1
**Cardiac Necrosis:**					
Normal (0, or 1)	21	19	23	25	12
Abnormal (2 or 3)	0	0	0	0	3
**Liver Fibrosis:**					
Normal (0, or 1)	21	18	23	25	12
Abnormal (2 or 3)	0	1	0	0	3
**Liver Necrosis:**					
Normal (0, or 1)	21	17	22	25	14
Abnormal (2 or 3)	0	2	1	0	1
**Protozoal Cysts:**					
Rare (none or rare)	13	9	7	5	2
Present (few, moderate, or severe)	8	10	16	20	13
